# Molecular characterization of extended spectrum cephalosporin resistant *Escherichia coli* isolated from livestock and in-contact humans in Southeast Nigeria

**DOI:** 10.3389/fmicb.2022.937968

**Published:** 2022-07-22

**Authors:** Solomon O. Olorunleke, Miranda Kirchner, Nicholas Duggett, Manal AbuOun, Onyinye J. Okorie-Kanu, Kim Stevens, Roderick M. Card, Kennedy Foinkfu Chah, John A. Nwanta, Lucy A. Brunton, Muna F. Anjum

**Affiliations:** ^1^Veterinary Epidemiology, Economics and Public Health Group, Department of Pathobiology and Population Sciences, Royal Veterinary College, London, United Kingdom; ^2^Department of Bacteriology, Animal and Plant Health Agency, Weybridge, United Kingdom; ^3^Department of Veterinary Public Health and Preventive Medicine, University of Nigeria, Nsukka, Enugu, Nigeria; ^4^Department of Animal Science, Ebonyi State University, Abakaliki, Nigeria; ^5^School of Health and Life Science, Teesside University, Middlesbrough, United Kingdom; ^6^Department of Veterinary Pathology and Microbiology, University of Nigeria, Nsukka, Enugu, Nigeria

**Keywords:** *Escherichia coli*, ESBL, AMR, cefotaxime, livestock, in-contact humans, Southeast Nigeria

## Abstract

The rise in antimicrobial resistance (AMR) in bacteria is reducing therapeutic options for livestock and human health, with a paucity of information globally. To fill this gap, a One-Health approach was taken by sampling livestock on farms (*n* = 52), abattoir (*n* = 8), and animal markets (*n* = 10), and in-contact humans in Southeast Nigeria. Extended spectrum cephalosporin (ESC)-resistant (ESC-R) *Escherichia coli* was selectively cultured from 975 healthy livestock faecal swabs, and hand swabs from in-contact humans. Antimicrobial susceptibility testing (AST) was performed on all ESC-R *E. coli*. For isolates showing a multi-drug resistance (MDR) phenotype (*n* = 196), quantitative real-time PCR (qPCR) was performed for confirmation of extended-spectrum β-lactamase (ESBL) and carbapenemase genes. Whole-genome sequencing (WGS) was performed on a subset (*n* = 157) for detailed molecular characterisation. The results showed ESC-R *E. coli* was present in 41.2% of samples, with AST results indicating 48.8% of isolates were phenotypically MDR. qPCR confirmed presence of ESBL genes, with *bla*_CTX-M_ present in all but others in a subset [*bla*_TEM_ (62.8%) and *bla*_SHV_ (0.5%)] of isolates; none harboured transferable carbapenemase genes. Multi-locus sequence typing identified 34 Sequence Types (ST) distributed among different sampling levels; ST196 carrying *bla*_CTX-M-55_ was predominant in chickens. Large numbers of single nucleotide polymorphisms (SNPs) in the core genome of isolates, even within the same clade by phylogenetic analysis, indicated high genetic diversity. AMR genotyping indicated the predominant *bla*_CTX-M_ variant was *bla*_CTX-M-15_ (87.9%), although *bla*_CTX-M-55_, *bla*_CTX-M-64,_ and *bla*_CTX-M-65_ were present; it was notable that *bla*_CTX-M-1_, common in livestock, was absent. Other predominant AMR genes included: *sul*2, *qnr*S1, *str*B, *bla*_TEM-1b_, *tet*A-v2, and *dfr*A14, with prevalence varying according to host livestock species. A *bla*_CTX-M-15_ harbouring plasmid from livestock isolates in Ebonyi showed high sequence identity to one from river/sewage water in India, indicating this ESBL plasmid to be globally disseminated, being present beyond the river environment. In conclusion, ESC-R *E. coli* was widespread in livestock and in-contact humans from Southeast Nigeria. WGS data indicated the isolates were genetically highly diverse, probably representing true diversity of wild type *E. coli*; they were likely to be MDR with several harbouring *bla*_CTX-M-15._ Surprisingly, human isolates had highest numbers of AMR genes and pigs the least.

## Introduction

Resistance to third-generation cephalosporins (e.g., cefotaxime and ceftazidime) by the production of extended-spectrum β-lactamase (ESBL) enzymes (mainly *bla*_CTX-M_, *bla*_TEM_, and *bla*_SHV_ genes; Louka et al., 2021) among *Enterobacteriaceae*, including *E. coli* has been identified as a critical emerging issue of public health concern by the World Health Organization (World Health Organisation, 2017). Commensal *E. coli* are classified as indicator organisms in the monitoring of antimicrobial resistance (AMR) spread in livestock as their spread in the food chain poses a risk to public health (Duggett et al., 2020). Globally, the *bla*_CTX-M_ gene has been more frequently detected among clinical *E. coli* isolates (Zorgani et al., 2017); hence, it has gained more prominence than the other β-lactamase genes such as *bla*_SHV_ and *bla*_TEM_. Much information has been gained on their presence and distribution across Europe, including the United Kingdom, through active monitoring of AMR in humans and livestock (European Centre for Disease Prevention and Control, 2017, Abuoun et al., 2020) due to surveillance programs that already exists. However due to globalization of travel and trade, these isolates are likely to be disseminated worldwide, making it important to determine their distribution globally.

Plasmids are the essential vectors in the horizontal transfer and dissemination of AMR genes in *Enterobacteriaceae* and have significantly contributed to the rise in AMR among *E. coli* strains (Xia et al., 2017). Several plasmid families (IncF, IncI1, IncI2, IncX, IncA/C, and IncHI2) have been identified to play a crucial role in the emergence and dissemination of ESBL-producing *Enterobacteriaceae* (Wang et al., 2018). The majority of them (e.g., IncF, IncA/C, IncN, IncHI2, and IncK) are also epidemic plasmids that are often detected among food animals and humans (Irrgang et al., 2017).

In the global context, there is currently a paucity of information from Africa, including Nigeria, on the prevalence of ESBL-producing *Enterobacteriaceae* and their circulating plasmids in livestock and humans, especially those in close contact with animals. For Nigeria, in particular Southeast Nigeria, the few reports on ESBL genes detected in *E. coli* are predominantly from studies of human clinical isolates from tertiary hospitals (Iroha et al., 2012; Nwafia et al., 2019) and to a lesser extent from animals (Chah et al., 2018). Therefore, this study aimed to fill a gap in our current knowledge on ESBL-producing *E. coli* present in livestock and humans in close contact, in Southeast Nigeria. The objective was to purify extended spectrum cephalosporin-resistant (ESC-R) *E. coli* from samples collected from healthy livestock faeces and in-contact humans in Abia, Ebonyi, and Enugu States in Nigeria, and to phenotypically determine resistance to a panel of antimicrobials. For a selected subset of MDR isolates, where the ESBL genotype had been verified by PCR, whole genome sequencing (WGS) was performed to determine the entire AMR gene profile. Multi-locus sequencing and phylogenetic reconstruction was undertaken to determine the genotypic diversity of these isolates. A *bla*_CTX-M-15_ gene bearing plasmid present in a small subset of ESBL *E. coli* was further characterized to determine how conserved it remained between isolates from different compartments, as well as to establish its identity to global AMR plasmids.

## Materials and methods

### Ethical statement

The methods/procedures used in this study were concomitant with that outlined in the Animals Scientific Procedures Act of 1986 for the care and use of animals for research purposes. An approval was obtained from the Research Ethics Committee of the Faculty of Veterinary Medicine, University of Nigeria, Nsukka (Approval Reference Number: FVM-UNN-IACUC-2019-0570).

### Sampling and isolation of bacteria

A two-stage cluster sampling was employed. First, we randomly selected 3/5 of the states in Southeast Nigeria (Abia, Ebonyi, and Enugu) by balloting and second, within the states, we collected samples from abattoirs (*n* = 8), animal markets (*n* = 10), and livestock farms (*n* = 52). The sample size was determined using the formula from previous descriptive studies (Araoye, 2008), and a minimum sample size of 384 samples was calculated. However, we collected a total of 975 samples during the raining and dry hot season. Faecal swabs were randomly collected from one out of every 10 apparently healthy livestock (maximum of 10 swabs were collected from cattle, chicken, goat, pig, and sheep per time), and non-probability convenience sampling was employed to take hand swabs from willing in-contact humans at each sampling site. The faecal (*n* = 755) and hand swab (*n* = 220) samples were first enriched in peptone broth over night before appropriate dilutions of the samples were plated on cefotaxime (5 μg/ml) supplemented MacConkey agar and incubated at 37°C for 18–24 h. One non-duplicate lactose fermenting colony was selected from each primary plate and sub-cultured on eosin methylene blue agar and incubated aerobically at 37°C for 18–24 h. Typical *E. coli* colonies were 2–3 mm in diameter, with little tendency to confluent growth, exhibiting a greenish metallic sheen by reflected light and dark purple centre by transmitted light. Such colonies were then further confirmed as *E. coli* using API20E kit. The total numbers of *E. coli* purified from each host species is provided in Supplementary Table S1.

### Antimicrobial susceptibility testing

Antimicrobial susceptibility testing (AST) of 14 antimicrobials (Supplementary Table S2) was performed on the presumptive ESC-R *E. coli* by disc diffusion, and the resistance breakpoint was determined according to the Clinical and Laboratory Standards Institute guidelines (Clinical and Laboratory Standards Institute, 2017). The ESBL phenotype of isolates were confirmed by sensitivity to amoxicillin/clavulanic acid but as some isolates also harboured Class C beta-lactamases some isolates with ESBL genes also showed resistance to amoxicillin/clavulanic acid.

### Detection of ESBL and carbapenemase genes

A subset of 196 ESC-R *E. coli* showing resistance to at least one agent in three or more classes of antimicrobials (i.e., MDR) phenotypically were screened for ESBL and carbapenemase genes (*bla*_CTX-M_, *bla*_SHV,_ *bla*_TEM,_ *bla*_OXA-48,_ *bla*_VIM,_ *bla*_NDM,_ and *bla*_KPC_) using two multiplex qPCRs (Supplementary Table S3). The methods previously described for identification of ESBL genes (Roschanski et al., 2014) were modified by the inclusion of *Enterobacteriaceae* 16S rRNA primers and probe while the methods previously described for carbapenemase genes (Van Der Zee et al., 2014) were also employed.

### Phylogenetic analysis and detection of CTX-M gene variants

Genomic DNA was extracted and WGS was performed on *bla*_CTX-M_ positive *E. coli* (*n* = 157) identified by qPCR using Illumina NextSeq platform and the APHA SeqFinder pipeline used to detect AMR genes, including *bla*_CTX-M_ gene variants (Duggett et al., 2017, Stubberfield et al., 2019, Abuoun et al., 2020). This subset also included isolates which phenotypically showed resistance to carbapenem. Kraken v2 was used to obtain an accurate taxonomic classification of the *E. coli* and the MLST profile identified (Duggett et al., 2017). For phylogenetic analysis, Snippy v3 was used to detect single nucleotide polymorphisms (SNPs; *n* = 140 raw reads that certify the inclusion criteria) against the reference genome (*E. coli* K12 MG1655). Areas of recombination were removed using Gubbins (Abuoun et al., 2020) and a core genome phylogenetic tree was constructed using RAxML with 100 bootstrap iterations performed to estimate confidence in clustering. We annotated the maximum likelihood phylogenetic tree with iTOL v3 (Duggett et al., 2020). Abricate was used to identify the plasmid replicons carrying *bla*_CTX-M_ gene in the genomes assembled using SPAdes v3.12.0 (Nurk et al., 2013), and annotated using BRIG, as previously described (Alikhan et al., 2011). A core genome pairwise SNP distance matrix was generated (Supplementary Table S4) using snp-dists v0.6.^1^ BlastN was used to detect the percentage identity between the genome of plasmid pAEB010 and pV234 (NCBI accession number LC056430.1). All WGS were deposited at ENA (study accession number: PRJEB43719).

## Results

### AMR phenotypes and β-lactamase confirmation

**Figure 1:**
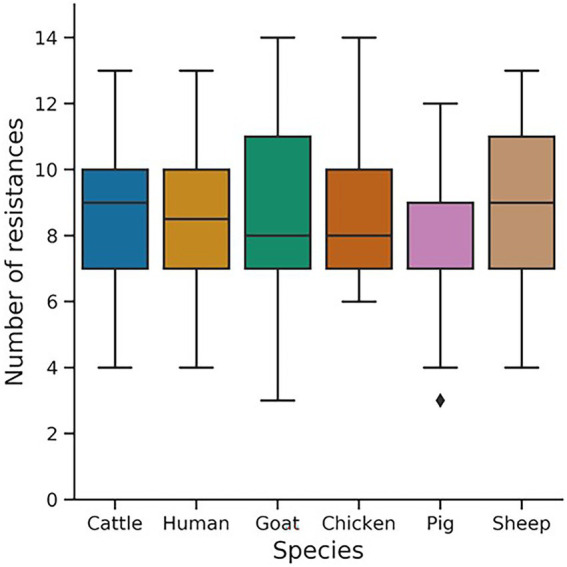
Box plot of the total number of resistances in *Escherichia coli* isolates (*n* = 402) from different livestock species and in-contact humans. The 14 antimicrobials used for the antimicrobial susceptibility test include Meropenem (10 μg), Ceftazidime (30 μg), Cefotaxime (30 μg), Aztreonam (30 μg), Ampicillin(10 μg), Amoxicillin/clavulanic acid (30 μg), Enrofloxacin (5 μg), Ofloxacin (5 μg), Norfloxacin (10 μg), Ciprofloxacin (10 μg), Gentamicin (10 μg), Streptomycin (5 μg), Sulphamethoxazole/Trimethoprim (25 μg), and Tetracycline (30 μg).

Using an antibiotic selective approach, ESC-R *E. coli* was detected in 41.2% (402/975) of samples collected randomly from different livestock host and in-contact humans in Southeast Nigeria. There were differences in the prevalence of ESC-R *E. coli* detected from the different sampling levels with the numbers isolated from cattle, chicken, goat, and sheep being 38.8% (85/219), 34.6% (56/162), 36.5% (54/152), and 56.0% (47/84), respectively; while those from human (44.1%, 97/220) and pigs (44.4%, 63/142) were similar. AST was performed on all 402 ESC-R *E. coli* to determine their resistance profile, and showed resistance to aztreonam, ampicillin, and cefotaxime. The levels of isolates showing resistance to streptomycin, tetracycline, sulfamethoxazole/trimethoprim, and ceftazidime ranged between 65 and 91%; but ≤40% of the isolates were resistant to amoxicillin/clavulanic acid, gentamicin, meropenem, and any of the quinolones/fluoroquinolones tested (Table 1). Almost half the *E. coli* (48.8%; 196/402) were phenotypically MDR, however, isolates from goats and chickens harboured the highest number of co-resistances (up to 14) compared to isolates from other species (Figure 1). Also, pig isolates harboured less co-resistances (on average seven, with the highest number being 12) than any other host species (Figure 1).

**Table 1:** Antimicrobial resistance profile of *Escherichia coli* isolates (*n* = 402) from livestock and in-contact humans in Southeast Nigeria.

Class of antimicrobial	Antimicrobial agent	Number (%) of isolates resistant
Carbapenem	Meropenem (10 μg)	114 (28)
Third-generation cephalosporin	Ceftazidime (30 μg)	364 (91)
	Cefotaxime (30 μg)	402 (100)
Penicillin	Ampicillin (10 μg)	402 (100)
Monobactam	Aztreonam (30 μg)	402 (100)
β-Lactam inhibitor	Amoxicillin/ clavulanic acid (30 μg)	159 (40)
Quinolone and fluoroquinolones	Ofloxacin(5 μg)	108 (27)
	Norfloxacin (10 μg)	115 (29)
	Ciprofloxacin (10 μg)	142 (35)
	Enrofloxacin (5 μg)	115 (29)
Aminoglycoside	Gentamicin (10 μg)	120 (30)
	Streptomycin (5 μg)	261 (65)
Folate pathway inhibitor	Sulfamethoxazole/ trimethoprim (25 μg)	316 (79)
Tetracycline	Tetracycline (30 μg)	305 (76)

Multiplex qPCR was used for detection of β-lactamases and confirmed presence of *bla*_CTX-M_ (100%, 196/196), accounting for the ESBL phenotype in all the isolates. Additionally, *bla*_TEM_ and *bla*_SHV_ were detected in 62.8% (123/196) and 0.5% (1/196) of isolates, respectively, but the carbapenemase genes (*bla*_OXA-48,_ *bla*_VIM,_ *bla*_NDM,_ and *bla*_KPC_) included in our qPCR panel were not detected in any isolate.

### Sequence types and phylogeny

**Figure 2:**
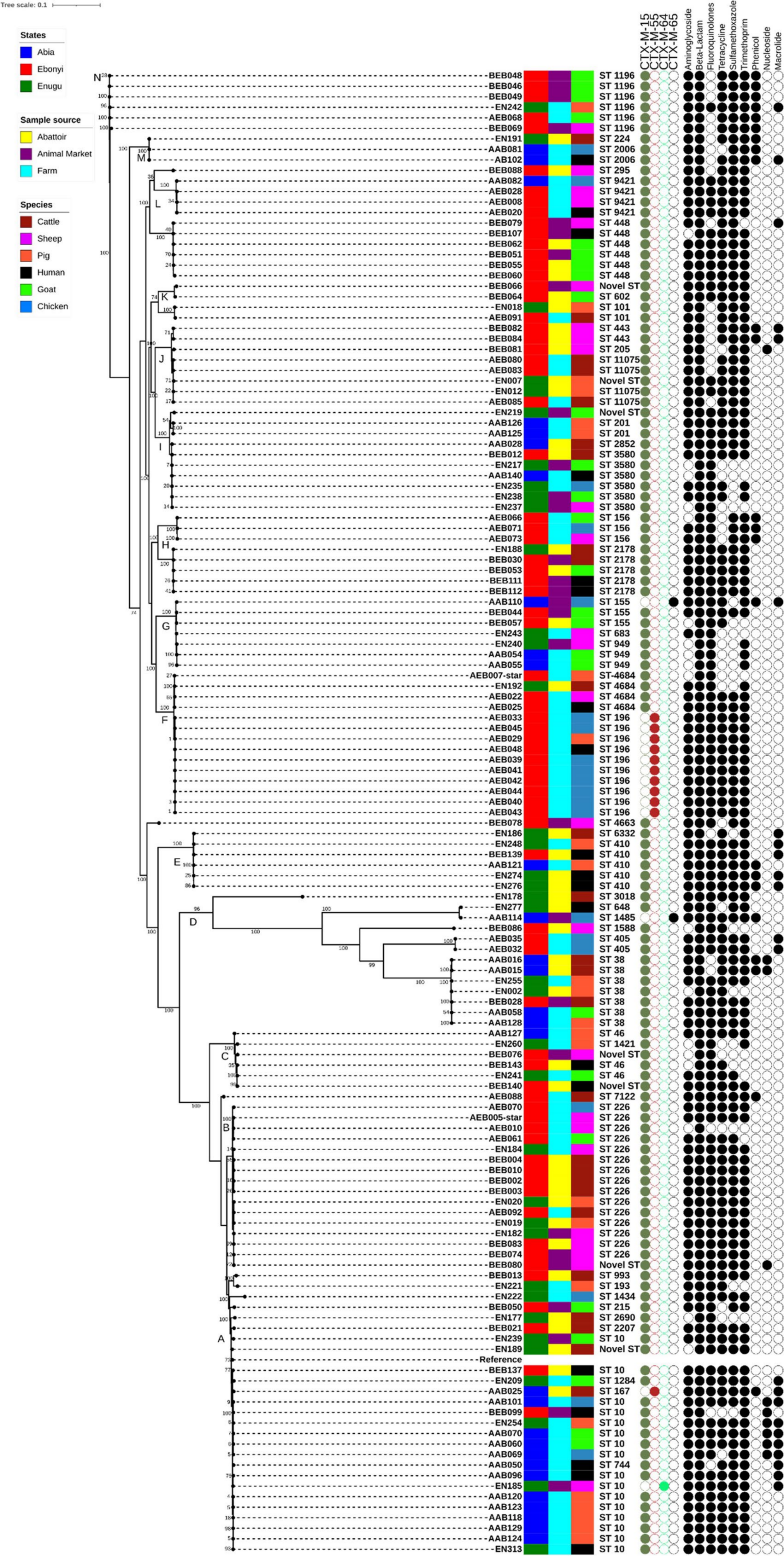
Maximum likelihood core-genome single nucleotide polymorphism (SNP) tree showing phylogenetic relatedness of 140 isolates from Southeast Nigeria. Genetic relatedness is indicated by branch length and position confidence in clades by bootstrap confidence values displayed on the tree. Also given are the multi-locus sequence type, the *bla*_CTX-M_ variant and MDR profile (black circles indicated presence of at least one resistance gene variant while the white circles indicated the absence of AMR genes conferring resistance to antimicrobials tested) of each *E. coli* isolate, along with the state, sample source, and host animal. *Escherichia coli* K-12 was used as the reference genome in building the tree.

*In silico* MLST analysis of 157 *E. coli* using the WGS data identified 34 different sequence types (STs; Figure 2) circulating within the livestock and in-contact human population in Southeast Nigeria, with ST10 and ST226 being the most predominant. Four STs (ST38, ST156, ST1196, and ST11075) were found in both monogastric and ruminants, while ST9421 was found in *E. coli* isolates from humans, monogastric and ruminant animals. Some STs (ST648, ST744, ST193, ST201, and ST1421) were present singly or in low numbers in specific hosts so their distribution could not be confirmed.

A core-genome SNP-based maximum-likelihood phylogenetic tree was constructed to reveal the phylogenetic relatedness of the host *E. coli*. It distributed isolates into 14 different clades, and there was generally a high level of confidence in the clades (A-N; Figure 2) due to high bootstrap values, which mostly ranged between 74 and 100%. Most isolates from the same ST clustered together, with a clade harbouring isolates from different sampling levels, i.e., states, sample source, and host species. This was especially evident in clade A, the predominant clade harbouring Clonal Complex 10 isolates with isolates from all three states and sample source, as well as the six host species present. In contrast isolates from ST196, present in clade F, were an exception; isolates from this ST were predominantly from chicken farms in Ebonyi State and not widely dispersed amongst different sampling levels. Core genome pairwise SNP analysis (Supplementary Table S4) showed *E. coli* from livestock and in-contact humans to be highly diverse with isolates from clade A having a median pairwise core genome SNP distance of 17,500 (range 80–65,766), indicating these were not the same clones transferring between compartments. Similarly, clade B and F isolates, predominantly ST226 and ST196, had median pairwise core genome SNP distances of 28,078 (range 83–28,252) and 1,209 (range 187–14,958), respectively; indicating clade F isolates were less diverse than in clade A and B but nevertheless quite far apart. Clade G isolates (comprising ST949, ST155, and ST683) had the lowest median pairwise core-genome SNP distance of 728 (range 276–14,112), despite belonging to different STs, with subclades harbouring isolates of the same ST showing a lower core genome SNP diversity (e.g., ST949, range 276–728). A similar core genome SNP diversity was noted in most other clades with mixed STs (e.g., H, I, J, L, and N), where subclades of same STs were less diverse, although still several hundred SNPs apart. It was notable that clade D, showing the longest branch lengths but a bootstrap confidence value of 96%, comprised a variety of STs (ST38, ST405, ST1588, ST648, and ST1485) with a median pairwise core-genome SNP distance of 72,865 (range 959–120,175) between isolates, indicating that although they were more distantly related to each other than in all clades, there was high confidence in the clustering together of these isolates (Figure 2).

### AMR genes and plasmids

Despite the genomic diversity of *E. coli* only 69 AMR genes were present in the different antimicrobial classes examined using APHA SeqFinder; none harboured any transferable gene encoding resistance to carbapenem. Within the ESBL category, four different variants of CTX-M genes (*bla*_CTX-M-15_, *bla*_CTX-M-55_, *bla*_CTX-M-64,_ and *bla*_CTX-M-65_) were detected through WGS (Figure 2), with *bla*_CTX-M-15_ being predominant (88%, 138/157). Examination of the distribution of different variants of *bla*_CTX-M_ among *E. coli* revealed that all *E. coli* isolates from goats and humans were *bla*_CTX-M-15_ carriers, which was also predominant in cattle, pigs, and sheep (93%; Table 2). However, only 48% of chicken isolates carried *bla*_CTX-M-15_; the remaining harboured different *bla*_CTX-M_ variants (*bla*_CTX-M-55,_ *bla*_CTX-M-64,_ and *bla*_CTX-M-65_). Other β-lactamases identified in this study included *bla*_TEM-1b_ (64.3%, 101/157), *bla*_OXA-1_ (10%, 15/157), *bla*_ACT-25_ (1.3%, 2/157), and *bla*_SHV-28_ (0.6%, 1/157).

**Table 2:** Antimicrobial resistance genes detected in ESBL *E. coli* isolates from livestock and in-contact humans in Southeast Nigeria.

Antimicrobial class	Antimicrobial resistance gene	Overall number of isolates harbouring gene (*N* = 157)	Distribution of AMR genes among species					
			Human (*n* = 18)	Cattle (*n* = 27)	Chicken (*n* = 23)	Goat (*n* = 29)	Pig (*n* = 28)	Sheep (*n* = 30)
Aminoglycoside	*str*B	104 (66.2)^*^	12 (66.7)	**22 (81.5)** ^**^	10 (43.5)	18 (62.1)	21 (75.0)	21 (70.0)
	*str*A	97 (61.8)	11 (61.1)	**22 (81.5)**	8 (34.8)	16 (55.2)	20 (71.4)	20 (66.7)
	*aac*3-IId	27 (17.2)	3(16.7)	2 (7.4)	**13 (56.5)**	3 (10.3)	4 (14.3)	2 (6.7)
	*aac*6-Ib-cr	18 (11.5)	**5 (27.8)**	1 (3.7)	2 (8.7)	2 (6.9)	4 (14.3)	4 (13.3)
	*aadA*5	16 (10.2)	**5 (27.8)**	0	3 (13.0)	2 (6.9)	4 (14.3)	2 (6.7)
	*aadA*2	13 (8.3)	1 (5.6)	3 (11.1)	3 (13.0)	**5 (17.2)**	0	1 (3.3)
	*ant*3-Ia	12 (7.6)	2 (11.1)	2 (7.4)	**3 (13.0)**	2 (6.9)	1 (3.6)	2 (6.7)
	*ant*3-1a	10 (6.4)	1 (5.6)	2 (7.4)	0	**5 (17.2)**	0	2 (6.7)
	*aadA*6	8 (5.1)	2 (11.1)	1 (3.7)	1 (4.3)	0	0	**4 (13.3)**
	*aac*3-pEK516	7 (4.5)	2 (11.1)	0	0	1 (3.4)	0	**4 (13.3)**
	*aph*3-Ia	6 (3.8)	1 (5.6)	0	**2 (8.7)**	2 (6.9)	1 (3.6)	0
	*aadA*22_b	5 (3.2)	1 (5.6)	**2 (7.4)**	0	0	0	2 (6.7)
	*aac*6-Ib4	3 (1.9)	0	0	1 (4.3)	0	0	**2 (6.7)**
	*aac*6-Ib9	3 (1.9)	0	0	1 (4.3)	0	0	**2 (6.7)**
	*aac*6-Ib_a	3 (1.9)	0	0	1 (4.3)	0	0	**2 (6.7)**
	*aadA*15	3 (1.9)	0	**1 (3.7)**	0	1 (3.4)	1 (3.6)	0
	*aadA*12	1 (0.6)	0	0	0	**1 (3.4)**	0	0
	*aadA*24	1 (0.6)	**1 (5.6)**	0	0	0	0	0
	*aac*6-Ib3	1 (0.6)	**1 (5.6)**	0	0	0	0	0
Extended-spectrum Cephalosporin	CTX-M-15	138 (87.9)	**18 (100)**	26 (96.3)	11 (47.8)	**29 (100)**	26 (92.9)	28 (93.3)
	OXA-1	15 (9.6)	**5 (27.8)**	1 (3.7)	2 (8.7)	2 (6.9)	3 (10.7)	2 (6.7)
	CTX-M-55	12 (7.6)	1 (5.6)	1 (3.7)	**8 (34.8)**	0	1 (3.6)	1 (3.3)
	CTX-M-65	2 (1.3)	0	0	**2 (8.7)**	0	0	0
	ACT-25	2 (1.3)	**1 (5.6)**	0	0	0	0	1 (3.3)
	CTX-M-64	1 (0.6)	0	0	**1 (4.3)**	0	0	0
	SHV-28	1 (0.6)	0	0	0	**1 (3.4)**	0	0
Penicillin	TEM-1b	101 (64.3)	12 (66.7)	**20 (74.1)**	10 (43.5)	21 (72.4)	18 (64.3)	20 (66.7)
	TEM-1c	2 (1.3)	0	0	**1 (4.3)**	0	0	1 (3.3)
	TEM-1	1 (0.6)	0	0	**1 (4.3)**	0	0	0
	TEM-191	1 (0.6)	0	0	0	**1 (3.4)**	0	0
	TEM-1a	1 (0.6)	0	0	**1 (4.3)**	0	0	0
Fluoroquinolone	*qnr*S1	106 (67.5)	10 (55.6)	21 (77.8)	17 (73.9)	17 (58.6)	**23 (82.1)**	18 (60.0)
	*qnr*S4	4 (2.5)	0	0	0	2 (6.9)	0	2 (6.7)
	*qep*A	4 (2.5)	0	0	0	**4 (13.8)**	0	0
	*qnr*S3	2 (1.3)	0	0	0	0	0	**2 (6.7)**
	*qep*A4	2 (1.3)	**1 (5.6)**	1 (3.7)	0	0	0	0
	*qnr*B1	2 (1.3)	**1 (5.6)**	0	0	0	0	1 (3.3)
	*oqx*A10	1 (0.6)	0	0	0	**1 (3.4)**	0	0
	*oqx*A8	1 (0.6)	0	0	0	**1 (3.4)**	0	0
	*oqx*B17	1 (0.6)	0	0	0	**1 (3.4)**	0	0
	*qep*A2	1 (0.6)	0	0	0	**1 (3.4)**	0	0
Tetracycline	*tet*-A-v2	97 (61.8)	9 (50.0)	18 (66.7)	17 (73.9)	17 (58.6)	**21 (75)**	15 (50)
	*tet*-AB	24 (15.3)	**6 (33.3)**	2 (7.4)	2 (8.7)	5 (17.2)	4 (14.3)	5 (16.7)
	*tet*-A	4 (2.5)	1 (5.6)	**2 (7.4)**	0	1 (3.4)	0	0
	*tet*-D	2 (1.3)	0	**2 (7.4)**	0	0	0	0
Sulfamethoxazole	*sul*2	115 (73.2)	14 (77.8)	**23 (85.2)**	18 (78.3)	18 (62.1)	21 (75.0)	21 (70.0)
	*sul*1	21 (13.4)	**7 (38.9)**	1 (3.7)	3 (13.0)	3 (10.3)	4 (14.3)	3 (10.0)
	*sul*3-v1	12 (7.6)	1 (5.6)	1 (3.7)	2 (8.7)	**6 (20.7)**	1 (3.6)	1 (3.3)
Trimethoprim	*dfr*A14	84 (53.5)	10 (55.6)	**19 (70.4)**	12 (52.2)	10 (34.5)	17 (60.7)	16 (53.3)
	*dfr*A17	20 (12.7)	**5 (27.8)**	0	4 (17.4)	6 (20.7)	4 (14.3)	1 (3.3)
	*dfr*A12	15 (9.6)	1 (5.6)	4 (14.8)	**4 (17.4)**	5 (17.2)	0	1 (3.3)
	*dfr*A1	13 (8.3)	1 (5.6)	2 (7.4)	3 (13.0)	2 (6.9)	1 (3.6)	**4 (13.3)**
	*dfr*2d	2 (1.3)	**1 (5.6)**	0	0	1 (3.4)	0	0
	*dfr*A8	2 (1.3)	0	**1 (3.7)**	0	0	1 (3.6)	0
	*dfr*B4	2 (1.3)	**1 (5.6)**	0	0	1 (3.4)	0	0
	*dfr*A8	1 (0.6)	0	**1 (3.7)**	0	0	0	0
	*dfr*A21	1 (0.6)	0	**1 (3.7)**	0	0	0	0
	*dfr*A22	1 (0.6)	0	**1 (3.7)**	0	0	0	0
Phenicol	*cat*A1	15 (9.6)	**4 (22.2)**	3 (11.1)	1 (4.3)	1 (3.4)	2 (7.1)	4 (13.3)
	*cml*_	10 (6.4)	1 (5.6)	2 (7.4)	1 (4.3)	**5 (17.2)**	0	1 (3.3)
	*cat*B3	3 (1.9)	0	0	1 (4.3)	0	0	**2 (6.7)**
	*flo*R	2 (1.3)	0	0	**2 (8.7)**	0	0	0
Phosphonic (Fosfomycin)	*fos*A	2 (1.3)	**1 (5.6)**	0	0	0	0	1 (3.3)
	*fos*A_v3	1 (0.6)	0	0	0	**1 (3.4)**	0	0
Macrolide	*mph*A	24 (15.3)	**6 (33.3)**	2 (7.4)	4 (17.4)	4 (13.8)	4 (14.3)	4 (13.3)
	*mef*B	5 (3.2)	0	0	**2 (8.7)**	2 (6.9)	1 (3.6)	0
	*erm*B	1 (0.6)	**1 (5.6)**	0	0	0	0	0
	*mph*B	1 (0.6)	0	0	**1 (4.3)**	0	0	0
Nucleoside	*sat*2A	10 (6.4)	1(5.6)	2 (7.4)	**2 (8.7)**	2 (6.9)	1 (3.6)	2 (6.7)

* Figures in brackets represent % of each cell.

** Figures in bold signifies the host species with highest percentage number of resistances.

The non-β-lactamase genes present in ESBL producing MDR *E. coli* are presented in Table 2. Among the 19 aminoglycoside resistance gene variants identified, *str*A (62%, 97/157) and *str*B (66%, 104/157) were predominant, especially among cattle isolates. Of the 10 plasmid-mediated quinolone resistance gene variants detected in this study, *qnr*S1 (68%, 106/157) was predominant, especially in isolates from pigs. On average, 62% (97/157) of the *E. coli* harboured a *tet*(A)-v2 gene, encoding resistance to tetracycline, predominantly in pig isolates. In contrast, *tet*(AB) was more prevalent in human isolates; while *tet*(A) and *tet*(D) were prevalent in cattle. The predominant sulfamethoxazole resistance gene was *sul*2. Approximately, 85% of cattle isolates harboured the *sul*2 gene while *sul1* and *sul3* were predominantly identified in human and goat isolates, respectively. Among the trimethoprim resistance genes identified, *dfr*A14 was predominant, especially in cattle isolates. Other AMR genes identified included those encoding resistance to phenicol, macrolide, nucleoside, and phosphonic resistances but none of the isolates harboured transferable carbapenemase genes, despite some showing phenotypic resistance. For these isolates the ResFinder pipeline (Bortolaia et al., 2020), and BLASTN from NCBI^2^ were also used but neither detected any carbapenem resistant genes (data not shown). Overall, the average number of AMR genes per isolate was highest in human isolates and lowest from pigs (Table 2).

**Figure 3:**
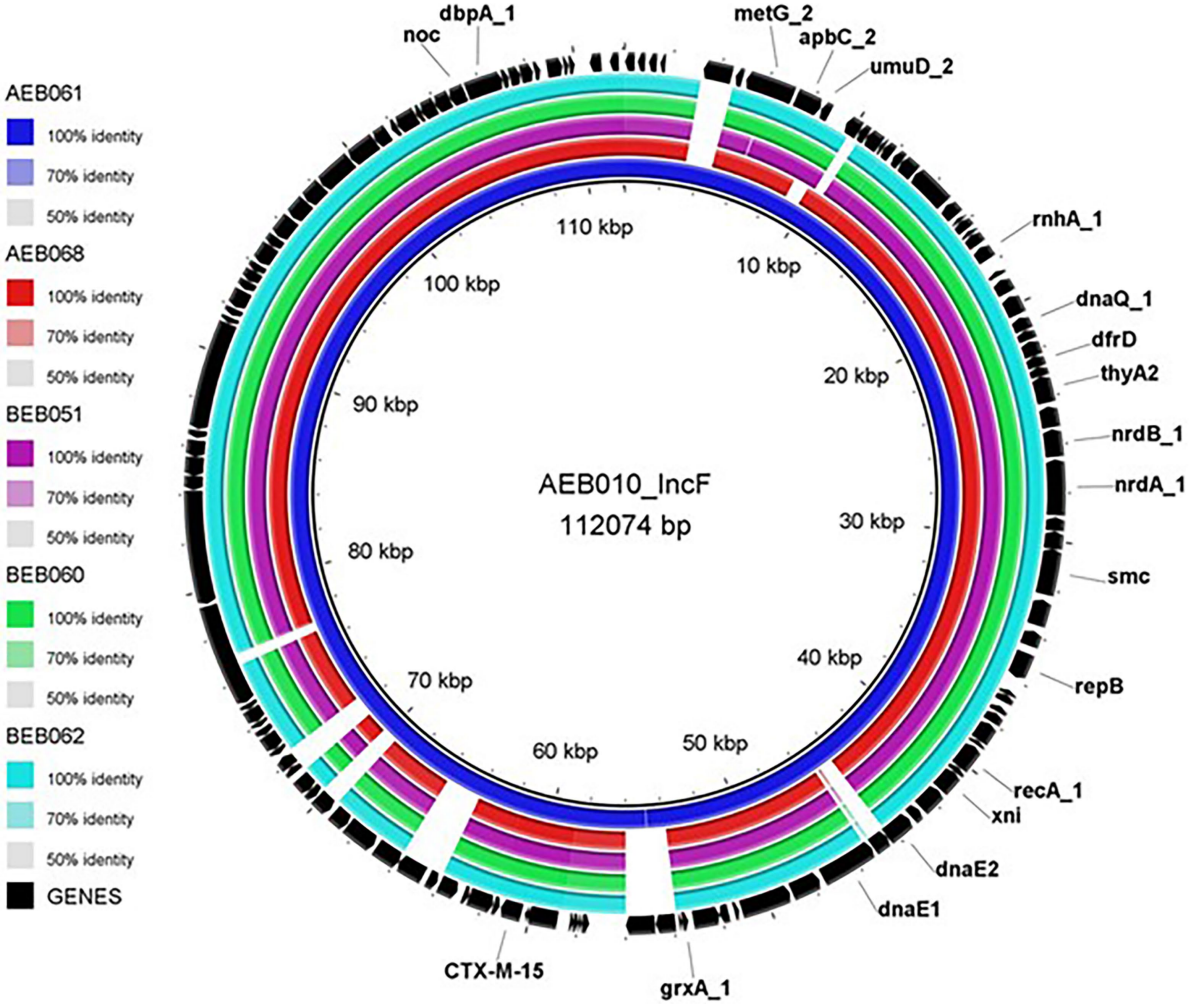
BRIG image comparing the IncFIB plasmid in AEB010 isolate with other *E. coli* isolates carrying the *bla*_CTX-M-15_ gene.

As described above although a high level of genetic diversity was observed among the *E. coli*, many harboured similar AMR genes. There were several isolates where AMR genes and plasmid replicon were identified in the same contig (Table 3). In total, 13 different plasmid replicon types were identified; however, only IncFIB, IncX, and IncY were identified to harbour *bla*_CTX-M_ variants. Isolates carrying IncFIB plasmid predominantly co-harboured *bla*_CTX-M-15_, and BRIG identified similarity between isolates carrying this plasmid (Figure 3). Size of the presumptive IncFIB plasmid (pAEB010) from a sheep *E. coli* isolate (AEB010) was 112,074 bp; it was 99.99% identical at the nucleotide level to a 112,009 bp ESBL plasmid isolated from an *E. coli* in India (Akiba et al., 2016). The contig containing IncFIB plasmid and *bla*_CTX-M-15_ from isolate AEB061 from goat was 100% identical to pAEB010 identified in a sheep isolate from the same farm. Other isolates (AEB068, BEB051, BEB060, and BEB062) also contained contigs which harboured pAEB010 but slightly differed, with several ORFs of unknown function deleted from the pAEB010-like plasmid in these isolates (Figure 3). It is interesting to note that the six pAEB010 harbouring isolates were from different STs, and clustered within various clades (Figure 2), suggesting plasmid transmission in these isolates, which were all from Ebonyi. Although isolates AEB010 and AEB061 were from sheep and goat host species respectively, they were from the same farm and isolated 3 months apart, suggesting the persistence of pAEB010 plasmid on the farm. It was interesting to identify similar pAEB010-like plasmids in isolates from abattoir (BEB060 and BEB062) and animal market (BEB051) samples, as these sites serve as a convergent area for livestock but mostly from the Northern part of Nigeria. All pAEB010-like plasmid harbouring isolates except AEB010 harboured eight AMR genes conferring resistance to aminoglycoside, cephalosporin, penicillin, fluoroquinolone, sulphonamide, tetracycline, and trimethoprim classes of antibiotics. The remaining AMR genes were not present on the same plasmid and require further investigation (Table 2).

**Table 3:** Plasmid replicon and the antimicrobial resistance (AMR) genes harboured in the same contig.

Isolate	State	Sample origin	Species	AMR genes (*n*)	Plasmid	Genes carried on the plasmid
AAB060	Abia	Farm	Goat	16	ColRNAI_1	*qnr*S1
AAB069	Abia	Farm	Chicken	11	ColRNAI_1	*aph*3-Ia + *sul*2
AAB083	Abia	Farm	Chicken	3	ColRNAI_1	*str*A + *str*B + *sul*2
AEB011	Ebonyi	Farm	Sheep	3	ColRNAI_1	*str*A + *str*B + *sul*2
AEB022	Ebonyi	Farm	Sheep	8	ColRNAI_1	*str*B + *bla*_TEM-1b_ + *dfr*A14 + *sul*2
AEB025	Ebonyi	Farm	Human	8	ColRNAI_1	*str*B + *bla*_TEM-1b_ + *dfr*A14 + *sul*2
AEB071	Ebonyi	Farm	Chicken	13	ColRNAI_1	*str*A + *str*B + *sul*2
AEB073	Ebonyi	Farm	Sheep	13	ColRNAI_1	*str*A + *str*B + *sul*2
BEB081	Ebonyi	Abattoir	Sheep	10	ColRNAI_1	*str*A + *str*B + *sul*2
BEB082	Ebonyi	Abattoir	Sheep	8	ColRNAI_1	*str*A + *str*B + *sul*2
BEB084	Ebonyi	Abattoir	Sheep	8	ColRNAI_1	*str*A + *str*B + *sul*2
EN209	Enugu	Farm	Goat	11	ColRNAI_1	*str*A + *str*B + *sul*2
EN277	Enugu	Abattoir	Human	9	ColRNAI_1	*str*B + *dfr*A14 + *sul*2
AEB080	Ebonyi	Farm	Cattle	7	IncFIB	*bla*_CTX-M-15_ + *int*2 + *qnr*S1
AEB083	Ebonyi	Farm	Cattle	7	IncFIB	*bla*_CTX-M-15_ + *int*2 + *qnr*S1
AEB010	Ebonyi	Farm	Sheep	1	**IncFIB**	*bla*_CTX-M-15_
AEB061	Ebonyi	Farm	Goat	8	**IncFIB**	*bla*_CTX-M-15_
AEB068	Ebonyi	Farm	Goat	8	**IncFIB**	*bla*_CTX-M-15_
BEB051	Ebonyi	Animal Market	Goat	8	**IncFIB**	*bla*_CTX-M-15_
BEB060	Ebonyi	Abattoir	Goat	8	**IncFIB**	*bla*_CTX-M-15_
BEB062	Ebonyi	Abattoir	Goat	8	**IncFIB**	*bla*_CTX-M-15_
BEB066	Ebonyi	Animal Market	Sheep	8	IncFIB	*bla*_CTX-M-15_ + *qnr*S1
AAB114	Abia	Animal Market	Chicken	9	IncFIC	*str*A + *str*B + *bla*_TEM-1b_ + *sul*2
AAB110	Abia	Animal Market	Chicken	9	IncFII	*bla*_TEM-1b_
EN235	Enugu	Farm	Chicken	6	IncFII	*bla*_TEM-1b_
AAB060	Abia	Farm	Goat	16	IncHI2_1	*aac*3-IId
BEB081	Ebonyi	Abattoir	Sheep	10	IncI1_1_Alpha	*aac*3-IId + *bla*_TEM-1b_
AEB068	Ebonyi	Farm	Goat	8	IncN_1	*aad*A2 + *dfr*A12 + *int*1 + *sul*3-v1
BEB046	Ebonyi	Animal Market	Goat	8	IncN_1	*aad*A2 + *dfr*A12 + *int*1 + *sul*3-v1
BEB049	Ebonyi	Animal Market	Goat	8	IncN_1	*aad*A2 + *dfr*A12 + *int*1 + *sul*3-v1
BEB069	Ebonyi	Animal Market	Sheep	8	IncN_1	*aad*A2 + *dfr*A12 + *int*1 + *sul*3-v1
BEB140	Ebonyi	Abattoir	Human	8	IncN_1	*int*1 + *dfr*A14
AAB050	Abia	Farm	Human	9	IncQ1_1	*str*B + *sul*2
AAB096	Abia	Farm	Human	10	IncQ1_1	*sul*2
AAB121	Abia	Farm	Pig	14	IncQ1_1	*str*A + *str*B + *sul*2
BEB051	Ebonyi	Animal Market	Goat	8	IncQ1_1	*str*A + *str*B + *sul*2
BEB055	Ebonyi	Abattoir	Goat	8	IncQ1_1	*str*A + *str*B + *sul*2
BEB060	Ebonyi	Abattoir	Goat	8	IncQ1_1	*str*A + *str*B + *sul*2
BEB062	Ebonyi	Abattoir	Goat	8	IncQ1_1	*str*A + *str*B + *sul*2
EN274	Enugu	Abattoir	Human	14	IncQ1_1	*str*A + *str*B + *sul*2
EN276	Enugu	Abattoir	Human	14	IncQ1_1	*str*A + *str*B + *sul*2
BEB048	Ebonyi	Animal Market	Goat	8	IncR_1	*aad*A2 + *int*1 + *dfr*A12 + *sul*3-v1
AAB025	Abia	Abattoir	Cattle	13	IncX1_1	*bla*_CTX-M-55_
AAB121	Abia	Farm	Pig	14	IncX3_1	*bla*_TEM-1b_ + *qnr*S1
BEB028	Ebonyi	Animal Market	Cattle	8	IncY_1	*bla*_CTX-M-15_ + *str*A + *str*B + *bla*_TEM-1b_ + *dfr*A14 + *int*1 + *qnr*S1 + *int*2 + *sul*2
BEB030	Ebonyi	Animal Market	Cattle	8	IncY_1	*int*2
BEB053	Ebonyi	Abattoir	Goat	8	IncY_1	*dfr*A14 + *int*1
EN003	Enugu	Abattoir	Pig	8	IncY_1	*int*2
EN219	Enugu	Animal Market	Goat	8	IncY_1	*int*2

Isolate harbouring a 112 Kb IncFIB blaCTX-M-15 plasmid is given in bold.

## Discussion

The presence of ESC-R *E. coli* with ESBL genes in the food chain poses a public health risk (Duggett et al., 2021). Although this is a global One-Health issue, the paucity of data worldwide makes it difficult to assess their distribution. To help fill the knowledge gap, in this study, we sampled livestock on farms and abattoir, as well as in-contact humans in three states of Southeast Nigeria, which demonstrated presence of ESC-R *E. coli* in 41.2% of samples, which nearly half, were phenotypically MDR; presence of an ESBL gene in these isolates was demonstrated by PCR. *bla*_CTX-M-15_, a predominant ESBL-producing gene among *E. coli* in human infections (Day et al., 2019), was predominant in the isolates genotyped by WGS, which were from different livestock species and in-contact humans, suggesting widespread dissemination of this variant within our sampling set. Our findings were similar to a previous report (Chah et al., 2018) on the predominance of *bla*_CTX-M-15_ among livestock, in Nsukka, Enugu and also to a study from Ghana showing presence of similar ESBL types from farm workers and livestock (Falgenhauer et al., 2018); other organisms such as livestock associated MRSA have long shown such association (Anjum et al., 2019). We also identified different variants of *bla*_CTX-M_ with predominance of *bla*_CTX-M-55_ in ST196 among chicken isolates in Ebonyi State, which to the best of our knowledge is the first report of *bla*_CTX-M-55_ from chicken in Nigeria; however, this variant was present in other host species including humans. Predominance of *bla*_CTX-M-55_ in ST196 among chicken isolates may be due to all chicken farms in Southeast Nigeria included in this study receiving their poults from the same hatchery in Southwest Nigeria. Although *bla*_CTX-M-55_ differs from *bla*_CTX-M-15_ by a single amino acid substitution, there has been increased isolation of this variant globally, especially in animals, after the first report from Thailand in 2006 (Hu et al., 2018), indicating a possible adaptation within animals. Likewise, *bla*_CTX-M-64_ and *bla*_CTX-M-65_ identified in chicken and in-contact human isolates had not previously been reported from Nigeria. Conversely, we noted absence of *bla*_CTX-M-1_, commonly associated with ESBL *E. coli* from livestock (Duggett et al., 2020), from all isolates genotyped in this study. This may be due to our selection method which used 5 μg/ml of cefotaxime, whilst others that have reported presence of *bla*_CTX-M-1_ have used 1 μg/ml of cefotaxime (Irrgang et al., 2018; Duggett et al., 2020).

Extended-spectrum β-lactamase-producing *E. coli* co-harboured AMR genes conferring resistance to other AMR classes, which differed by animal host. For example, isolates from pigs predominantly harboured fluoroquinolone (*qnr*S1) and tetracycline (*tet*(A)-v2) resistance. The decline of fluoroquinolone usage in pigs in Europe is recognised (Bennani et al., 2020), but there are no available data on their use in Nigeria, which could be moderate to high and promote resistance in *E. coli*. In contrast, tetracycline is frequently used in the livestock industry in Nigeria (Adesokan et al., 2015). Although the hands of in-contact humans may be contaminated with livestock bacteria, we noted host-specificity of human isolates for tetracycline [*tet*(AB)] and sulfamethoxazole (*sul*1). Cattle isolates had a higher percentage harbouring aminoglycoside (*strA* and *strB*), sulfamethoxazole (*sul*2), and trimethoprim (*dfr*A14) resistance genes. These antimicrobials are commonly used by farmers in treatment of cattle diarrhoea in Nigeria (Alhaji and Isola, 2018). Analysis of WGS data indicated isolates showing resistance to meropenem phenotypically did not harbour any known transferable carbapenemase genes; thus, the mechanism of meropenem resistance in this study remained unclear. Discrepancies between phenotypic and genotypic resistances, as observed in this study, have been previously reported (Heyckendorf et al., 2018; Doyle et al., 2020; Urmi et al., 2020) and could be due to novel mechanisms or overexpression of an efflux pump that require future investigation.

Isolates belonging to the same ST generally clustered together suggesting some possible links between geographic location, sample source, and host species. However, large SNP distances in the core genome demonstrated that isolates even of the same ST were highly diverse, with the most closely related isolates being several hundred SNPs apart, suggesting they were unlikely to be the same clones and may represent the true genetic diversity that exists within wild type *E. coli.* These results are unlike those found from WGS based studies on, for example, United Kingdom pig farms where *E. coli* of the same ST were usually more closely related (Abuoun et al., 2020; Duggett et al., 2020), with those <14 SNPs apart in the whole genome being described as the same clone (Storey et al., 2022). These differences could be due to the livestock pyramid breeding system that exists in UK, which may promote dissemination of more closely related *E. coli* through the host animal, not being so widespread in Southeast Nigeria.

Although isolates were genetically highly diverse, we noted concordance in AMR genotypes which indicated possible circulation of common AMR plasmids. Of the 13 different plasmid replicon types identified to be co-present with AMR genes, only three contained *bla*_CTX-M_ variants; further characterization using long read WGS will help identify these plasmids more fully in future. Nevertheless, we identified pAEB010 which showed >99% identity to an ESBL plasmid from *E. coli* isolated from river/sewage water in India (Akiba et al., 2016). This plasmid and close variants were detected in highly diverse sheep and goat isolates with large SNP distances in the core genome, from the same farm sampled several months apart, and furthermore the same plasmid was found in isolates from an abattoir, and animal market in Ebonyi, where these animals are expected to be slaughtered and sold subsequently. This demonstrated transmission between these compartments in Southeast Nigeria of what may be a highly mobile, stable and persistent global plasmid harbouring *bla*_CTX-M-15_. Also, the *bla*_CTX-M-15_ samples bearing pAEB010 from this study are likely to represent only a small proportion of isolates positive for this plasmid on the farms, abattoir, and animal market from Ebonyi due to limitations in the numbers of samples collected per location.

The presence and dissemination of MDR *E. coli* in Nigeria may be associated with selective pressure from the use of antimicrobials without prescription, especially in livestock (European Food Safety Authority; European Centre for Disease Prevention and Control, 2017) and could be a source of human infections (Abuoun et al., 2020; Wee et al., 2020), although direct transmission between animal and human compartments was not evident. However, cefotaxime and other cephalosporins are not commonly used in management of livestock diseases in Southeast Nigeria due to high costs. Hence presence of ESC-R *E. coli* among livestock was probably due to past transmission from in-contact humans to animals, where it proliferated and then circulated further. Although for several of the ESC-R *E. coli* that were MDR, it could be due to co-selection from use of other antimicrobials on farm. While this study was limited to food animals and in-contact humans, the roles of the environment and wild birds in the dissemination of AMR harbouring *E. coli* could also be a key factor (Singer et al., 2016; Gweon et al., 2019; Stanton et al., 2020; Anjum et al., 2021; Storey et al., 2022), which requires future examination, especially as pAEB010 was highly similar to a plasmid present in *E. coli* from water sources in India, and its route of transmission between these distal places remains unknown.

In conclusion, four different variants of *bla*_CTX-M_ were identified among the ESC-R *E. coli* from livestock and humans in Southeast Nigeria. Although there was a high level of genetic diversity amongst the *E. coli* many harboured similar AMR genes, and a global ESBL plasmid present in isolates from different sampling levels demonstrated this may be due to widespread mobile genetic elements transmission. Long read sequencing in future will help verify other common ESBL plasmids circulating not only in isolates from this study but to those reported globally.

## Limitation of this study

The sample collection and phenotypic characterisation phase of the study was carried out with limited resources within a short time frame in Nigeria and hence constraining the antibiotics susceptibility testing of the *E. coli* isolate to only the antibiotic discs available in University of Nigeria, Nsukka. Although we identified meropenem-resistant isolates phenotypically, we could not assess the susceptibility to ertapenem due to limited resources which could not fund additional work. Furthermore, we identified a high level of genetic diversity instead of clonal transmission in our dataset, however, we could not perform additional conjugation assay to confirm the ability of the plasmids to spread as there was not enough time and resources.

## Data availability statement

The datasets presented in this study can be found in online repositories. The names of the repository/repositories and accession number(s) can be found in the article/Supplementary Material.

## Author contributions

SOO, KFC, JAN, LAB, and MFA contributed to conception and design of the study. MK, ND, MA, RMC, and OJO-K organized the dataset. SOO, KS, and LAB performed the statistical analysis and interpretation. SOO wrote the first draft of the manuscript. KFC, LAB, and MFA wrote sections of the manuscript. All authors contributed to the article and approved the submitted version.

## Funding

This research was mainly funded by the Commonwealth Scholarship Commission in the United Kingdom and additional funding was from the Royal Veterinary College, University of London, and the UK FAO Reference Centre for Antimicrobial Resistance (which receives funding from the Department for Environment, Food and Rural Affairs and UK aid funding from the Department of Health and Social Care’s Fleming Fund).

## Conflict of interest

The authors declare that the research was conducted in the absence of any commercial or financial relationships that could be construed as a potential conflict of interest.

## Publisher’s note

All claims expressed in this article are solely those of the authors and do not necessarily represent those of their affiliated organizations, or those of the publisher, the editors and the reviewers. Any product that may be evaluated in this article, or claim that may be made by its manufacturer, is not guaranteed or endorsed by the publisher.

## Footnotes

^1^ https://github.com/tseemann/snp-dists

^2^ https://blast.ncbi.nlm.nih.gov/
