# Supplementary material for: Molecular characterization of extended spectrum cephalosporin resistant Escherichia coli isolated from livestock and in-contact humans in Southeast Nigeria
Source: Front Microbiol. 2022 Jul 22;13:937968. doi: 10.3389/fmicb.2022.937968 (PMC9354541; doi:10.3389/fmicb.2022.937968)
Supplement: Supplementary file 2 [file Data_Sheet_1.docx]

**Molecular characterization of cefotaxime-resistant *Escherichia coli* isolated from livestock and in-contact humans in Southeast Nigeria**

Solomon O. Olorunleke^1, 2, 4, 6^, M. Kirchner^2^, N. Duggett^3^, M. AbuOun^2^, O. J. Okorie-Kanu^4^, K. Stevens^1^, R.M. Card^2^, K. F. Chah^5^, J.A Nwanta^4^ L. A. Brunton^1^, and M. F. Anjum^2^*

**Supplementary Tables**

**Table S1.** Samples collected from each species and the total number of *E. coli* purified.

| **Species** | **Total number of Samples Collected N=975** | **Total number of *E. coli* purified from CTX supplemented MacConkey agar (%)** |
| --- | --- | --- |
| Human | 220 | 102 (25.4%) |
| Cattle | 210 | 83 (20.6%) |
| Chicken | 157 | 58 (14.4%) |
| Goat | 155 | 52 (12.9%) |
| Pig | 152 | 61 (15.2%) |
| Sheep* | 81 | 46 (11.4%) |

*The population of sheep in Southeast Nigeria is generally low compared to other livestock.

**Table S2.** **Antimicrobial Susceptibility Testing** (Antibiotics used in the study and their resistance breakpoint)

| Class of Antimicrobial | Antimicrobial | Resistance breakpoint* (mm) |
| --- | --- | --- |
| Carbapenem | Meropenem (10µg) | **≤ 19** |
| Third-generation cephalosporin | Ceftazidime (30µg) | **≤ 17** |
|  | Cefotaxime (30µg) | **≤ 22** |
| Penicillin | Ampicillin (10µg) | **≤ 17** |
| Monobactam | Aztreonam (30µg) | **≤ 13** |
| β-Lactam inhibitor | Amoxycillin/ clavulanic acid (30µg) | **≤ 13** |
| Quinolone and fluoroquinolones | Ofloxacin(5µg) | **≤ 12** |
|  | Norfloxacin (10µg) | **≤ 12** |
|  | Ciprofloxacin (10µg) | **≤ 15** |
|  | Enrofloxacin(5µg) | **≤ 12** |
| Aminoglycoside | Gentamicin (10µg) | **≤ 12** |
|  | Streptomycin(5µg) | **≤ 11** |
| Folate pathway inhibitor | Sulfamethoxazole/ trimethoprim (25µg) | **≤ 10** |
| Tetracycline | Tetracycline (30µg) | **≤ 11** |

**Table S3-a. Real-Time PCR primers and probes and conditions for identification of the presence of ESBL and carbapenemase genes.**

RT-PCR Primer and Probe Sequences

| **Gene** | **Primer Sequence 5’ – 3’** | **Reference** |
| --- | --- | --- |
| TEM | Forward GCATCTTACGGATGGCATGA  Reverse GTCCTCCGATCGTTGTCAGAA  Probe [6FAM] CAGTGCTGCCATAACCATGAGTGA [BHQ1] | * |
| SHV | Forward TCCCATGATGAGCACCTTTAAA  Reverse TCCTGCTGGCGATAGTGGAT  Probe [Cyanine5] TGCCGGTGACGAACAGCTGGAG [BHQ2] |  |
| CTX-M | Forward ACCGAGCCSACGCTCAA  Reverse CCGCTGCCGGTTTTATC  Probe [Cyanine3] CCCGCGYGATACCACCACGC [BHQ2] |  |
| 16S-RNA | Forward CCTCTTGCCATCGGATGTG  Reverse GGCTGGTCATCCTCTCAGACC  Probe [TexasRed] GTGGGGTAACGGCTÇACCTAGGCGAC [BHQ2] |  |
| OXA-48 | Forward GCGTGGTTAAGGATGAACAC  Reverse CATCAAGTTCAACCCAACCG  Probe [6FAM] AGCCATGCTGACCGAAGCCAATG [BHQ1] | ** |
| VIM | Forward GAGATTCCCACGCAYTCTCTAGA  Reverse AATGCGCAGCACCAGGATAG  Probe [HEX] ACGCAGTGCGCTTCGGTCCAGT [BHQ1] |  |
| NDM | Forward CATTAGCCGCTGCATTGATG  Reverse GTCGCCAGTTTCCATTTGCT  Probe [Cyanine5] CATGCCCGGTGAAATCCGCC [BHQ2] |  |
| KPC | Forward TGCAGAGCCCAGTGTCAGTTT  Reverse CGCTCTATCGGCGATACCA  Probe [Cyanine3] TTCCGTCACGGCGCGCG [BHQ2] |  |

*Roschanski N, Fischer J, Guerra B. *et al*. Development of a Multiplex Real-Time PCR for the Rapid Detection of the Predominant Beta-Lactamase Genes CTX-M, SHV, TEM and CIT-Type AmpCs in Enterobacteriaceae. *PLoS One* 2014; **9:** e100956

**van der Zee A, Roorda G, Bosman G. *et al.* Multi-centre evaluation of real-time multiplex PCR for detection of carbapenemase genes OXA-48, VIM, IMP, NDM and KPC. *BMC Infect. Dis.* 2014; **14**: 27.

**Table S3-b**. RT- PCR Reaction mixture for 16S, CTX-M, TEM and SHV genes

| RT-PCR MIX | Volume |
| --- | --- |
| Qiagen 2x multiplex master mix (QuantiTect Multiplex PCR) | 12.5 µl |
| Multiplex primer equimolar mix (CTX, TEM, SHV- 1µl each of forward and reverse primers at conc. 10pmol) | 6 µl |
| 0.4 µl each of 16S forward and reverse primer (conc. 10µM) | 0.8 µl |
| 0.1 µl of TEM Tag Man probe (conc. 5pmol) | 0.1 µl |
| 0.2 µl of each of CTX and TEM probes (conc. 5pmol) | 0.4 µl |
| 0.2 µl of 16S probe (conc. 1 µM) | 0.4 µl |
| RNase free water | 4 µl |
| DNA template | 1 µl |
| Total volume for reaction | **25 µl** |

**Table S3-c**. RT-PCR conditions for 16S, TEM, SHV and CTX-M Reaction

| Cycling Temperature | Time | Number of Cycles |
| --- | --- | --- |
| 95^0^C | 15 min | Preliminary heating |
| 95^0^C | 30 sec | **30 Cycles** |
| 50^0^C | 90 sec |  |
| 70^0^C | 60 sec |  |

**Table S3-d.** RT- PCR Reaction mixture for OXA-48, VIM, NDM and KPC genes

| OXA-48, VIM, NDM and KPC RT-PCR | Volume |
| --- | --- |
| Qiagen 2x multiplex master mix (QuantiTect Multiplex PCR) | 10 µl |
| Multiplex primer equimolar mix (OXA-48, VIM, NDM and KPC – 0.6 µl each of forward and reverse primers at conc. 0.75µM) | 4.8 µl |
| 0.375 µl each of OXA-48 and VIM probe (conc. 0.1875 µM) | 0.75 µl |
| 0.5 µl of NDM and KPC probe (conc. 0.25µM) | 1 µl |
| RNase free water | 2.45 µl |
| DNA template | 1 µl |
| Total volume for reaction | **20 µl** |

**Table S3-e.** RT-PCR conditions for blaOXA-48, blaVIM, blaNDM and blaKPC genes

| Cycling Temperature | Time | Number of Cycles |
| --- | --- | --- |
| 95^0^C | 15min | Preliminary heating |
| 95^0^C | 15sec | **50 Cycles** |
| 60^0^C | 60sec |  |
